# Current views of community and hospital pharmacists on pharmaceutical care services in the United Arab Emirates: A mixed methodological study

**DOI:** 10.12688/f1000research.110102.2

**Published:** 2022-12-01

**Authors:** Zelal Kharaba, Joviana Farhat, Bassam S. Mahboub, Manal Ali Buabeid, Yassen Alfoteih, Yaser Al-Worafi, Ammar Jaber, Mohammad AlAhmad

**Affiliations:** 1 Department of Clinical Pharmacy, College of Pharmacy, Al Ain University, Abu Dhabi, 112612, United Arab Emirates; 2 AAU Health and Biomedical Research Center, Al Ain University, Abu Dhabi, 112612, United Arab Emirates; 3 Honorary Associate lecturer, Faculty of Medical Sciences, Newcastle University, Newcastle Upon Type, UK; 4 College of medicine and health sciences, Khalifa University, Abu Dhabi, 127788, United Arab Emirates; 5 Department of Respiratory Medicine, Rashid Hospital, Dubai Health Authority, Dubai, 4545, United Arab Emirates; 6 Medicalty and Health Sciences, Pharmacy and Pharmaceutical Sciences, Fatima College of Health Sciences, Abu Dhabi, United Arab Emirates; 7 Department of Dental Surgery, City University College of Ajman, Ajman, 18484, United Arab Emirates; 8 College of Pharmacy, University of Science and Technology of Fujairah, Fujairah, 2202, United Arab Emirates; 9 Department of Clinical Pharmacy & Pharmacotherapeutics, Dubai Pharmacy College for Girls, Dubai, 19099, United Arab Emirates; 10 Department of Clinical Pharmacy, College of Pharmacy, Al Ain University, Al Ain, 64141, United Arab Emirates

**Keywords:** hospital pharmacists, community pharmacists, pharmaceutical care services, interprofessional education.

## Abstract

**Background: **The profession of pharmacy has evolved significantly in recent years in terms of professional service delivery. The aim of this study was to explore the current views of pharmacists in the United Arab Emirates (UAE) on pharmaceutical care services and the nature of barriers encountered in practice using qualitative and quantitative assessment methods.

**Methods:** A cross-sectional study was conducted among hospital and community pharmacists (n = 305) between March and May 2021, using qualitative and quantitative assessment methods. In the qualitative phase, 15 interviews were conducted to explore five main criteria: patient information, inadequate patient counseling, prescribing errors prevention and identifying drug-related problems, lack of participation in health awareness programs, and barriers to pharmaceutical care implementation. In the quantitative phase, 305 consenting pharmacists completed a questionnaire on seven criteria: demographic profile, pharmacist-physician interaction, patient counseling assessment, patient reports of adverse drug events, pharmacist participation in health awareness programs, perceptions of reducing prescribing errors and identifying drug-related problems, and barriers to appropriate pharmaceutical care implementation.

**Results:** The results of both the qualitative and quantitative phases of the study revealed that pharmacists' influence on practice in the UAE is limited due to many factors, mainly lack of time and patients' ignorance of the pharmacist's role in the medical field. The mean responses regarding pharmacists' approach to patient counseling and patients' knowledge of pharmacists' role in managing adverse drug reactions were 77.1% and 59.7%, respectively. Active participation in health awareness programs was 64.8%. The mean positive response of participants in reducing prescribing errors and recognizing drug-related problems was 9.2%. Pharmacists' age and number of years in practice were the most important factors influencing the pharmaceutical care services implementation.

**Conclusion:** The study has shown the need to shed light on the proper implementation of pharmaceutical care while maintaining a trusting relationship with physicians.

## Introduction

Traditionally, pharmacy was considered a profession overlapping the health and chemical sciences, ensuring the safe use of medicines. Over time, the role of the pharmacist shifted from a product-oriented practice to a more patient-centered practice, giving rise to the concept of pharmaceutical care. Pharmaceutical care is the direct, responsible provision of medication-related care aimed at achieving specific outcomes and improving a patient’s quality of life. ^1^ Accordingly, the interaction between pharmacists, physicians, and other health care professionals has been influenced. Indeed, a close working relationship between pharmacists and physicians is critical to optimizing patient care. Thus, the study of pharmacy has changed globally over the past 30 years and aims to enhance the medical skills acquired during the professional career. Communication skills, experience and vigilance are the main internal factors that influence performance. On the other hand, environmental factors such as availability of restrooms are important external factors that improve the work of pharmacists.

Nowadays, pharmacists work in many different settings such as communities, hospitals, industrial environments and healthcare service institutions. ^2^ Consequently, continuous improvement of pharmacists’ experience and knowledge is the most important asset to achieve the highest possible level of professional execution through standardized goals that allow detailed access to drug therapy, optimization of patient outcomes, minimization of redundancies and interruptions in health care delivery, and increase of collaboration and synergy between pharmacists, physicians, and other health care professionals. ^3^

The above achievements have given pharmacy practice a social impact as basic and pre-licensure education provides pharmacists with broad knowledge and expertise on all aspects of preparation, distribution, action and use of drugs and medicines. ^4^

Therefore, a sufficiently stored scientific discipline of mind enabled them to be efficient self-learners. Although pharmacists are now able to fill their role in the community, they are still underestimated in the Middle East, especially in the United Arab Emirates (UAE), as their role as one of the main chief contributors in the treatment, consultation and follow-up of patients is somehow ignored. A recently published study investigated the impact of clinical and non-clinical risk factors on pharmacists’ performance and practice of pharmacy in public pharmacies in Abu Dhabi. This study reported a large gap in medication errors and computer system malfunctions, followed by theft incidents, and violent behavior that manifested in verbal, psychological, and physical assaults. ^5^

From a statistical perspective, the distribution of pharmacists may vary from city to city, reflecting the shortage and uneven distribution of health professionals. On the other hand, other critical obstacles also contribute to the limitation of pharmacy practice, namely: demographic factors such as the aging of the population and the geographical distribution of people, economic factors mainly related to the increased health care costs and the growing gap between wealthy and poor people, and sociological factors such as the use of traditional medicines even in severe cases. ^6^

In developing countries, the role and influence of pharmacists in society is still unclear and underappreciated. In a 2014 study on the role of pharmacists in the United Arab Emirates, the authors noted a number of challenges and barriers to optimizing pharmacy services. ^7^ These could be due to a lack of resources to compensate and retain pharmacists in community and hospital pharmacies, overlapping responsibilities of healthcare professionals, and a lack of interprofessional collaboration.

In practice, it is difficult to achieve ideal interprofessional collaboration in health care because it is difficult to demonstrate that all members of the health care team are equally responsible for and committed to achieving a particular clinical goal. It appears that physicians take the dominant role as clinical leaders in the hierarchical health care system, which may be related to the difficulties in creating a collaborative work environment when the pharmacist is considered a member of lesser importance and significance. ^8^ The result of this non-collaborative environment is a lack of coordination, sharing of information, experiences, publications, and research between pharmacists and other health professionals, leading to inadequate teamwork that impacts patient health. In addition, universities lack autonomy, governance, and institutional performance. ^9^

In addition, there is a lack of linkage between pharmaceutical implementation and national sustainable development plans. In several countries, the implementation of pharmaceutical care still faces a number of obstacles. Chief among these are lack of time, limited access to patients’ medication records, unsuitable premises, an insufficient number of competent pharmacists and the absence of standard practice strategies. ^10^^–^^13^

For this reason, this study will shed light on the current situation of pharmacy practice in the UAE while providing healthcare professionals with a suitable future-oriented work plan to help this profession become more influential in society.

## Methods

### Study design

This study was performed based on the (STROBE) Guidelines for Strengthening the Reporting of Observational Studies in Epidemiology for reporting cross-sectional observational studies and the Consolidated Criteria for Reporting Qualitative Studies (COREQ).

The study was conducted among hospital and community pharmacists across all emirates in the UAE between March and May 2021, using quantitative and qualitative assessment methods. In the quantitative phase, a paper-based questionnaire was used and distributed among different hospital and community pharmacists by the research team. In the qualitative phase, interviews were conducted among pharmacists who agreed and consented to enroll in the interview phase of the study.

### Study population

A total of 400 questionnaires were distributed to pharmacists. Of these, 305 pharmacists agreed to participate and completed the questionnaire (response rate 76.3%), 36 of whom were hospital pharmacists and 269 of whom were community pharmacists. Inclusion criteria required that participants were licensed pharmacists working in community or hospital pharmacies in the UAE for at least two years. Pharmacy technicians, non-licensed pharmacists, assistants or interns were excluded from this study.

Based on the previous literature and Raosoft sampling calculator, 300 responses for the quantitative phase and 15 for the qualitative phase were considered representative for this study.

### Ethics and consent

The study received the required ethical approval from the Research Ethics Committee (REC) of Al-Ain University (AAU-REC -B3, Feb 2021). Informed verbal consent was obtained prior to data collection for the anonymous use and sharing of participant data.

### Study tool

The questionnaire was designed after reviewing several literature reviews from previous studies. Accordingly, Murtaza G *et al*.’s (2015) questionnaire was used in this study. ^14^ Murtaza G *et al*.’s (2015) questionnaire was designed after a comprehensive review of literature and tested for validity and reliability. ^14^ We adopted this questionnaire and modified it slightly to suit the purpose and population of the present study. This questionnaire was completed individually by all participants. The research assistants were also available to answer or explain the participants’ questions and minimize the possibility of missing information.

The questionnaire consisted of seven criteria: 1) vital statistics of the population, 2) pharmacist-physician interactivity, 3) pharmacist’s role and involvement in patient counseling, 4) handling and reporting of adverse drug reactions in patients, 5) participation in health awareness programs, 6) pharmacist’s role in minimizing prescribing errors and identifying drug-related problems, 7) barriers to implementing pharmaceutical care in the UAE.

### Data collection

A non-probability convenient sampling technique was used for data collection due to the low willingness of pharmacists to complete the questionnaire and cooperate in the study. Participants underwent two phases of the study: quantitative and qualitative.

The quantitative phase focused on data collection through the distribution of a questionnaire using a non-systematic random sampling technique in all major cities of the UAE: Abu Dhabi, Dubai, Sharjah, Ajman, Umm Al Quwain, Ras Al Khaimah and Fujairah. The administered questionnaire was through a paper based survey with eligible pharmacists who agreed to participate in the study. The questionnaires were distributed by four research assistants who received a lecture on the topic and training on how to complete the questionnaire, all of which were conducted by one of main researchers (ZK). The research staff randomly approached eligible pharmacists in different community and hospital pharmacies in all emirates of the UAE. Participants were first informed of the purpose of the study and the estimated time required to complete the questionnaire. They were then given the choice of completing the questionnaire themselves or being supervised by a member of the study staff. Pharmacists were also informed of the anonymity and confidentiality of the study.

In the qualitative phase, a pilot interview was conducted. Participants were randomly selected and interviewed using a snowball approach. Participants were selected from the pool of participants from the quantitative phase. Only participants who agreed to be interviewed were considered in this phase of the study. The researcher (ZK) made an appointment with the participants by telephone and explained the aims and rationale for this research before conducting the interviews. Interviews were conducted face-to-face in hospital or community pharmacies for 30 minutes alone with (ZK), who is a female pharmacist and holds a PhD in clinical pharmacy and Good Clinical Practice (GCP) certificate. The saturation point was reached after 15 field note interviews. Field notes were completed during the interviews and discussed with the participants before they were finalized.

### Statistical analysis

Statistical Package for Social Sciences (SPSS version 21) was used for data entry and analysis. Descriptive analysis was used to record frequencies and percentages. A Chi-square test was used to determine the significant association between the dependent variables (pharmacists’ role and involvement in patient counseling, handling and reporting adverse effects of medications in patients, participation in health awareness programs, and pharmacists’ role in minimizing prescribing errors and identifying drug-related problems) and the independent variables (age and gender, type of pharmacy, and years of practice). A *p*-value of ≤ 0.05 was considered statistically significant at a 95% confidence interval for the differences.

## Results

In both qualitative and quantitative phases of the study, the age of participants ranged from 20 to 50 years, with a small number (n = 5) of older participants who were over 50 years old, as shown in Table 1, which presents the demographic profile of participants. No missing data were reported. In the qualitative phase, the thematic content analysis of the data obtained coded five main concerns, namely (a) patients reporting adverse drug events, (b) lack of patient counseling, (c) lack of participation in health awareness programs, (d) limitation in minimizing prescribing errors and identifying drug-related problems by pharmacists, (e) limitation in implementing pharmaceutical care services.

**Table 1.** Demographic profile of respondents.

Characterization	Quantitative, n (%)	Qualitative, n (%)
Age range (years)		
*20-30*	130 (42.7%)	6 (40%)
*31-40*	128 (41.9%)	3 (20%)
*41-50*	42 (13.8%)	3 (20%)
*Above 50*	5 (1.6%)	3 (20%)
Gender		
*Male*	146 (47.9%)	12 (80%)
*Female*	159 (52.1%)	3 (20%)
Nationality		
*Arabs*	177 (58%)	9 (60%)
*Non-Arabs*	128 (42%)	6 (40%)
Type of pharmacy		
*Community*	269 (88.2%)	9 (60%)
*Hospital*	36 (11.8%)	6 (40%)
Years of practice		
≥ *2-5*	136 (45%)	3 (20%)
*>5-10*	99 (33%)	3 (20%)
*>10-30*	64 (21%)	9 (60%)
*>30*	6 (1%)	0
Emirates		
*Sharjah*	101 (33.1%)	9 (60%)
*Dubai*	14 (4.6%)	6 (40%)
*Ajman*	97 (31.8%)	0
*Abu Dhabi*	86 (28.2%)	0
*Ras Al Khaimah*	3 (1%)	0
*Fujairah*	3 (1%)	0
*Umm Al Quwain*	1 (0.3%)	0

### Concern 1: Patients reporting adverse drug events

Adverse drug reactions are among the negative consequences of therapy, as they affect the quality of life and well-being of patients. A large proportion of patients are admitted to hospital due to adverse drug reactions. A significant portion of this morbidity and mortality can be prevented through the use of pharmaceutical care. Almost all participants felt that patients did not inform pharmacists of adverse drug events. The following are participant responses relating to this concern:

Community Pharmacist 1 **(CP1) said** “Because many patients do not know exactly what the pharmacist’s responsibilities are”

Patients are not fully aware of pharmacists’ duties and trust physicians to discuss patient complaints because physicians are the first people called when patients feel unwell or have complaints.

**CP3 said** “Patients do not inform the pharmacist because they do not even know that these effects are due to the drug itself”

Most patients are unable to distinguish whether their symptoms are related to the medications they have been taking or to their disease or disease progression.

Hospital Pharmacist 2 **(HP2) said** “Sometimes patients come to the hospital and buy many medicines together without informing us about the side effects they are facing. We try to minimize this by informing them about the interactions between the drugs they buy and see if we can help them”

Patients with polypharmacy are at high risk of side effects or drug interactions, most of which they are unable to recognise. They are unaware of this and require their physicians to change their prescriptions, usually without consulting the pharmacist to determine if there are specific interactions with other medications, foods, or even incorrect administration strategies.

### Concern 2: Lack of patient counseling

The pharmacist’s responsibilities include efficiently counseling and educating the patient, suggesting the best medications, and showing the patient how to get the most benefit from therapy. As part of pharmaceutical care, the pharmacist must educate patients on the proper use, dosage, administration, side effects, drug interactions, and storage of medications. This includes respect for the patient, empathy, maintaining privacy during consultations, good communication skills, prompt dispensing, and providing all drug information. The Lack of pharmacists’ involvement in patient counseling is the greatest limitation to the pharmacist’s counseling role, which is considered the mainstay of his or her practice, and also contributes to his or her social underestimation.

**CP2 said** “Most patients get advice from their doctor”

Physicians play an important role in patient counselling and education. Patients feel that they get what they need from their physicians, which has a significant impact on the pharmacist’s role in counselling.

**CP6 said** “They do not fully understand the pharmacist’s role as a medicines expert and are more likely to trust the doctor”

Patients want physicians to give them more medical advice about their conditions. They feel that this is what they wanted in the first place and that they are covered by their insurance and doctors should provide this for them.

**HP1 said** “Issues of regulation and legislation, e.g. we are not allowed to dispense medicines without a doctor’s prescription or authorisation”

The influence of health authorities and regulations on the role of the pharmacist in the treatment of self-limiting diseases with over-the-counter medications (OTC) and the other diseases is based on prescription medications (POM) administered by physicians, where the role of the pharmacist is to dispense only the approved prescriptions.

### Concern 3: Lack of participation in health awareness programs

Pharmacists play an important role in society. They are responsible for educating the public and spreading awareness about the rational use of medicines, as well as advising people about healthy diet and lifestyle. Such a role can be achieved through active participation in health education programs. Most pharmacists suffer from the fact that they rarely participate in such programs compared to other medical professions.

**CP4 said** “Because of the regulations in the medical institutions that do not allow pharmacists to play their role”

Pharmacists believe medical facilities are more actively involved in health education programs and invite more physicians than pharmacists.

**CP8 said** “Because of workload, lack of time and professional constraints”

In fact, there are several obstacles that limit the expansion of pharmacists’ activities outside their pharmacies, such as workload and staff shortages.

**HP6 said** “Time management is important because most pharmacists are busy managing cases and inpatients”

In the hospital, pharmacists spend more time managing and using medications, which in turn affects their ability to pursue other activities. In fact, hospital pharmacists should have time to participate in continuing education programs.

### Concern 4: Limitation in minimizing prescribing errors and identifying drug-related problems by pharmacists

Medications today are complex not only in pharmacological terms, but also in terms of their administration and dispensing, as they undergo a long process ranging from prescription by the physician, dispensing and counseling by the pharmacist, and use by the patient or caregiver. Because of this complexity, there is a risk of medication errors or drug-related problems. Therefore, the role of pharmaceutical care is to minimize and prevent these errors. In most cases, pharmacists use their expertise in medications in conjunction with ongoing direct consultation with the physician to maximize patient benefit and safety by avoiding prescribing errors and identifying drug-related problems as much as possible.

**CP9 said** “Pharmacists play a key role in reducing errors by reviewing prescriptions for drug interactions, dosing errors, contraindications, and other medication-related considerations”

Pharmacists are well positioned to play an important role in minimizing medication errors by reviewing medications prior to dispensing through a variety of processes, including double-checking strategy, patient reconciliation review, and prescription review based on the patient’s medical history.

**CP5 said** “By properly counseling and informing patients about drug-food interactions”

Identification of drug-related problems and minimization of errors can be achieved through appropriate consultation between the patient and the pharmacist. Several points can be emphasized, such as the correct strategy and timing for taking medications, avoiding drug-food interactions, or drug-drug interactions.

**HP4 said** “By calling the doctor and asking if they have provided the prescribed medication about the patient’s condition and if we can switch to a better medication”

In hospitals, communication between pharmacists and physicians can help achieve better patient outcomes by addressing untreated conditions and missing diagnoses and providing better options based on medications available in the hospital pharmacy.

**HP3 said** “In this hospital, we report any error by putting it in writing and sharing it with the whole team at the end of each month so that each of us is aware of any possible error”

Hospital pharmacists effectively participate in reporting medication errors by going through several steps, namely: consistently reporting errors, collecting the reports monthly, analyzing the reports based on the nature of the errors, providing solutions on how to avoid them in the future, then sharing and discussing the errors and solutions in meetings with the hospital medical team to raise awareness and prevent such errors.

### Concern 5: Limitations in implementing pharmaceutical care services

The implementation and adoption of pharmaceutical care can face many obstacles and barriers, such as lack of time due to workload, an insufficient number of qualified staff, lack of technology or skills in using technology programs, insufficient clinical and therapeutic knowledge, difficulty in accessing the patient’s medication and medical history, lack of communication skills, and lack of self-confidence. Other barriers include inadequate understanding of pharmaceutical care concepts and lack of patient and physician acceptance of pharmacist interventions. These barriers impede widespread adoption of this practice.

Pharmacists have been unable to develop a comprehensive practice due to numerous factors that continue to obscure their critical role and contribution to patient health.

**CP7 said** “The role of the pharmacist is being replaced by other healthcare providers”

Pharmacists believe that several tasks that pharmacists can perform have been replaced or are already covered by nurses and physicians. We believe that other health care providers can participate in pharmaceutical care through interprofessional collaboration, but qualified pharmacists are best suited to assume these roles.

**CP2 said** “Lack of opportunities for pharmacists”

Pharmaceutical care services are already widely used in health care systems. But it is not enough that these services exist; they should also be operated by qualified pharmacists. Therefore, appropriate positions for pharmacists should be created to successfully activate these services.

**HP5 said** “Market’s needs, depends on what are the markets needs at each different place and time.”

Some pharmacists believe that the implementation of pharmaceutical care is influenced by economics and markets. Such thoughts could be debunked if pharmaceutical care were properly implemented and presented as a strategy for cost-effective medication use. This leads to minimizing errors, using effective medications, and reducing drug costs, all of which have a major impact on hospital budgets.

In the quantitative phase, a total of 400 questionnaires were distributed. Of these, 305 pharmacists agreed to participate and completed the questionnaire (response rate 76.3%). The majority of the participants were between 20-40 years old (n = 258, 84.6%). The respondents were female (n = 159, 52.1%) and male (n = 146, 47.9%) pharmacists. More than half of the participants were Arab pharmacists (n = 177, 58%) and 42% (n = 128) were non-Arabs. Most participants worked in community pharmacies (n = 269, 88.2%) and to a lesser extent in hospital pharmacies (n = 36, 11.8%). Almost half of the participants (n = 136, 45%) had been practicing as pharmacists for less than five years but more than two years, while the others (n = 169, 55%) had been working as pharmacists for more than five years. Participants were from the seven largest cities in the UAE, Sharjah (n = 101, 32.9%), Ajman (n = 97, 31.8%), Abu Dhabi (n = 86, 28.2%), Dubai (n = 14, 4.6%), Ras Al-Khaimah (n = 3, 1%), Fujairah (n = 3, 1%) and Umm Al Quwain (n = 1, 0.3%). Table 1 shows the demographic profile of the respondents.

Pharmacists’ responses to the questions describing their interaction with physicians regarding patients’ medication showed different types of responses ( Table 2). The majority of pharmacists (n =139, 46%, n = 135 43%) responded “sometimes” or “rarely-never” to the question of whether physicians consider their suggestions and input regarding medication therapy. When asked how often they were contacted by physicians regarding their patients’ prescriptions, they responded “rarely to never” (n = 179, 58.69%). The most frequently cited reasons for these contacts were drug alternatives (n = 194, 63.5%), drug availability (n = 168, 55.1%), and drug dosage (n = 152, 49.8%). Most pharmacists (n = 153, 50%) responded “sometimes” when physicians considered their suggestions about drug-related problems. Table 2 shows the interaction with physicians regarding patients’ medication.

**Table 2.** Interaction with doctors on patients’ medication.

Variable	n (%)
Doctors call for advice	
Rarely-never	135 (43%)
Sometimes	139 (46%)
Always	31 (11%)
Contact with doctors regarding patients’ prescription	
Rarely-never	44 (14.42%)
Sometimes	179 (58.69%)
Always	82 (26.89%)
Reasons for interaction	
Drug availability	168 (55.10%)
Side effects	68 (20.90%)
Drug alternative	194 (63.50%)
Drug dosage	152 (49.80%)
Drug interactions	71 (23.30%)
ADRs	46 (15%)
Bad hand writing	6.4 (2.10%)
Pharmacists’ suggestions regarding drug related problems are considered by the physicians	
Never	80 (26%)
Sometimes	153 (50%)
Always	72 (24%)

Patient counseling is as important as drug therapy and is considered a major component of pharmaceutical care practice. When pharmacists were asked if physicians were willing to accept their involvement in patient counseling, 43.6% (n = 133) responded positively while 56.4% (n = 172) responded negatively. There was a significant relationship between physicians’ willingness to accept pharmacists’ involvement in patient counseling and pharmacists’ years in practice ( *p* < 0.001).

The majority of the pharmacists participated in patient education (n = 300, 98.6%), took adequate time for each patient (n = 246, 80.6%), informed patients about drug-drug or drug-food interactions (n = 240, 78.6%), instructed patients on the use of their medications (n = 282, 92.6%), informed patients about the side effects of medications (n = 200, 65.6%), and informed patients about proper storage conditions of medications (n = 243, 79.6%). The mean response regarding pharmacists’ approach to patient counseling was 77.1%. There was a significant association between the amount of time they spent with each patient and the type of pharmacy ( *p* = 0.0083), education about side effects, and years of pharmacist experience ( *p* = 0.0237). Table 3 illustrates the approach to patient counseling and pharmacists’ responses. To detail the patient counselling approach ( Table 3), there was a significant association between the amounts of time spent with each patient in community pharmacy compared to the hospital pharmacy. Additionally, pharmacists who practiced more than 10-years are significantly more engaged in patient counselling which reflect a higher physician acceptance rate as well as more patient education about side effects.

**Table 3.** Patient counseling approach.

Items in questionnaire	Responses		P value			
	Yes, n (%)	No, n (%)	Age	Gender	Type of pharmacy	Years of practice
Physicians are ready to accept your participation in patient counseling	133 (43.6%)	172 (56.4%)	0.506	1.000	0.7991	**<0.001**
Involved in educating patients	300 (98.6%)	5 (1.4%)	0.1970	0.1758	0.0771	0.7838
Spend enough time on each patient	246 (80.6%)	59 (19.4%)	0.6133	0.2645	**0.0083**	0.3119
Inform about drug and food interactions	240 (78.6%)	65 (21.3%)	0.3501	0.0562	0.6725	0.2489
Instruct patients in how to use their medications	282 (92.6%)	23 (7.4%)	0.6476	0.4700	0.7516	0.4579
Inform patients about drugs side effects	200 (65.6%)	105 (34.4%)	0.4594	0.9047	0.5807	**0.0237**
Inform patients about drugs storage conditions	243 (79.6%)	62 (20.4%)	0.1320	0.6761	0.3653	0.7378
Average	**234.9 (77.1%)**	**70.1 (22.9%)**				

The current study also found that 59.7% (n = 182 “mean”) of participants reported that their patients were aware of the pharmacist’s role in managing adverse drug reactions (ADRs). There was a significant association between the reported adverse drug reactions, pharmacist age ( *p* = 0.0216) and type of pharmacy ( *p* = 0.0482). Table 4 shows the patients’ reports of adverse drug reactions that occurred and the pharmacists’ responses. In detail, the community pharmacists showed significantly a higher level of patients’ awareness of adverse drug reaction reporting than hospital pharmacists.

**Table 4.** ADRs encountered patients’ reports.

Items in questionnaire	Responses		P value			
	Yes, n(%)	No, n(%)	Age	Gender	Type of pharmacy	Years of practice
Patients inform you about ADRs occurrence and ask about how it can be managed	191 (62.6%)	114 (37.4%)	**0.0216**	0.9066	0.2024	0.4222
Patients are aware of ADRs reporting	173 (56.6%)	132 (43.4%)	0.0995	0.1372	**0.0482**	0.4299
Average	**182 (59.7%)**	**123 (40.3%)**				

Participants were also asked about their active participation in health awareness programs and their responses were positive on average (n = 197.75, 64.8%). There was a significant relationship between health awareness participation ( *p* = 0.0441), pharmacists’ age ( *p* = 0.0002) and years in practice ( *p* = 0.042). Table 5 shows the participation of participants in health awareness programs. In details, there was a significant difference among the older age pharmacists and the participation in health awareness program and the organization in such program. In addition, pharmacists who have more years of experience showed more satisfaction toward the health awareness programs.

**Table 5.** Participation in health awareness programs.

Items in questionnaire	Responses		P value			
	Yes, n(%)	No, n(%)	Age	Gender	Type of pharmacy	Years of practice
Hospital organizes health awareness programs.	157 (51.6%)	148 (48.4%)	**0.0441**	0.6471	0.1112	**0.0051**
Your participation in organization of such programs.	89 (29.3%)	216 (70.7%)	**0.0002**	1.0000	0.8434	0.1307
Your satisfaction with these health awareness programs.	240 (78.6%)	65 (21.4%)	0.8519	0.2743	1.0000	**0.0420**
Pharmacists are encouraged to participate in such health awareness programs.	305 (100%)	0	N/A *	N/A *	N/A *	N/A *
Average	**197.75 (64.8%)**	**107.25 (35.2%)**				

* N/A: “not applicable”: it can’t be calculated.

Interestingly, the positive average of participants was in terms of reducing prescribing errors and identifying drug-related problems (n = 28, 9.2%). There was a significant association between finding irrelevant drug prescriptions and pharmacist age ( *p* = 0.0484). Table 6 shows participants’ perceptions of reducing prescribing errors and detecting drug-related problems. In details, pharmacists with more years of practice showed to be more active in identifying any irrelative drug prescription.

**Table 6.** Perception regarding reducing prescribing errors and identifying drug-related problems.

Items in questionnaire	Responses		P value			
	Yes, n (%)	No, n(%)	Age	Gender	Type of pharmacy	Years of practice
Find the drug interaction problems.	28 (9.2%)	277 (90.8%)	0.4686	0.5458	0.3421	0.1763
Find dose adjustment problems.	28 (9.2%)	277 (90.8%)	0.9044	0.1121	0.1096	0.1390
Find the irrelevant drug prescriptions.	28 (9.2%)	277 (90.8%)	**0.0484**	0.0678	0.3506	**0.0060**
Average	**28 (9.2%)**	**277 (90.8%)**				

**Figure 1.**
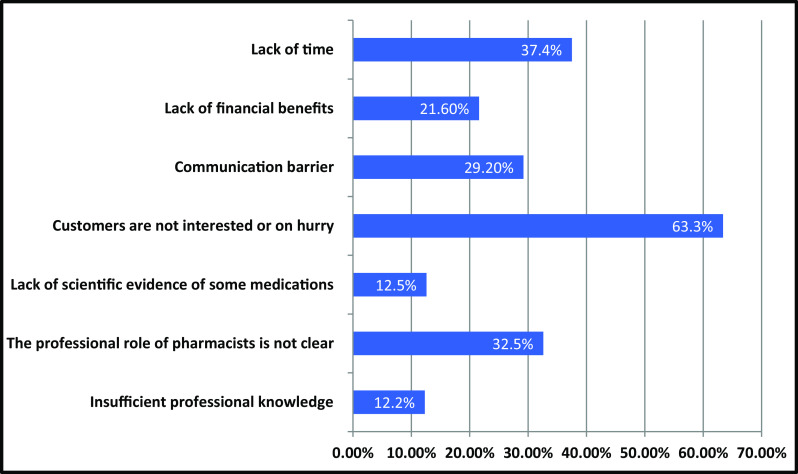
Barriers that prevents pharmaceutical care implementation.

The main barriers to implementing pharmaceutical care in the UAE cited by participants in this study were: Patients are not interested or in a hurry (n = 193, 63.3%), lack of time (n = 114, 37.4%), the professional role of pharmacists is not identified or clear (n = 99, 32.5%), communication barriers (n = 89, 29.2%), lack of financial benefits (n = 66, 21.6%), lack of scientific evidence for some medicines (n = 38, 12.5%), and inadequate expertise (n = 37, 12.2%). Figure 1 shows the barriers that prevent pharmacists from practicing pharmaceutical care for their patients.

## Conclusions/Discussion

Pharmaceutical care is considered a new concept in the UAE. This concept has only recently been introduced in pharmacy schools. Therefore, the adaptation of pharmaceutical care practice is still in its infancy.

Both the qualitative and quantitative phases of the current study showed almost comparable results: Pharmacists are not convinced that their suggestions and contributions as a member of the health care team are taken into account by physicians. This type of discrepancy affects trust between the two professions and impacts pharmacists’ performance when they make suggestions because they feel their input is ignored. The study also found that there is a lack of communication between physicians and pharmacists regarding drug information, despite physicians’ recognition of pharmacists’ role as drug experts. ^15^^,^^16^

In fact, the current results were consistent with several previous studies conducted in many Middle Eastern countries, including the UAE. ^17^^,^^18^ A nationwide survey of final year pharmacy students in the UAE on attitudes and perceived barriers related to pharmaceutical care showed that the majority of pharmacists believe in the importance of pharmaceutical care services in improving patient health and advancing pharmacy practice. However, the role of the pharmacist is still poorly perceived and the lack of a consultation area or inappropriate pharmacy design were cited as the main barriers. ^17^ In another study, the constant occurrence of medication dispensing errors was cited as a reason for introducing safe medication dispensing training programs. ^18^

Another study pointed to the fundamental development of strategies to improve the quality of pharmacy services, aimed at supporting the maximum number of pharmacies, increasing the frequency of patient consultations and reducing the number of treatment errors. ^14^

The consultative part of the interaction between the pharmacist and the patient is considered the core statement of pharmaceutical care practice. Thus, in his daily practice, the pharmacist must use his clinical knowledge, skills and attitude to provide the patient with a complete picture that includes information about the disease and every aspect of drug therapy, such as the use of the drug, its side effects, its interactions and storage conditions. A considerable number of pharmacists in this study are involved in patient counseling, but there are several barriers that prevent pharmacists from achieving a 100% counseling rate.

This may explain the lack of public confidence and education campaigns about the contribution of pharmacists to improving patient and public health. Given the limited statistical evaluation of such services in practice, the gradual introduction of an interprofessional education system in schools of medicine, pharmacy and nursing is a must. This will improve the existence of each medical profession in society while providing comprehensive and efficient patient-centred care. The impact of this new vision will contribute to an improved integration of pharmacists into the healthcare system. ^19^^–^^21^

According to the respondents in this study, not all patients are aware of the role of pharmacists in resolving adverse drug events, which is a barrier to the implementation of pharmaceutical care. Although pharmacists have been shown to share the most up-to-date evidence-based information from international sources such as the World Health Organization (WHO), the US Pharmacists Association, and the US Occupational Safety and Health Administration, and prevent crucial medication misuse or accidental loss. ^22^^–^^26^

Indeed, pharmacists have increased their knowledge of drugs and their side effects. At the same time, society chose pharmacists as the first direct source of up-to-date, trustworthy, and unbiased information. This frequent communication between pharmacists, patients and other health professionals strengthened the bond between individuals at the level of respect, trust, psychological support, information sharing and, above all, belief in the knowledge of each member as a dependent factor for the success of the whole scientific sector and the improvement of patient care. ^26^^,^^27^

Health awareness programs are sometimes organized in hospitals but not in pharmacies, so a large proportion of pharmacists cannot participate in these programs. Almost 50% of pharmacists who participated in this study cannot participate in such programs because their workplace does not organize health awareness programs. 100% of the participants agreed that pharmacists should participate more in health awareness programs because it will help the society to know the role of pharmacists and thus strengthen their position in the health care system and develop the pharmacy profession in this country.

Although this study found only a small percentage (9.2%) of positive perceptions in reducing prescribing errors and identifying drug-related problems, prescribing errors can be reduced if we use pharmacists’ drug knowledge and if pharmaceutical care practices are implemented correctly. Screening and correction of prescribing errors are considered one of the roles of pharmacists. ^28^^–^^30^

The majority of the participants in this study (63.3%) believed that patients are either not interested in pharmaceutical care services or are in a hurry, so pharmacists let patients leave the pharmacy without introducing them to the idea of pharmaceutical care and without giving the patient enough information about their medications. These incorrect assumptions are due to the pharmacists’ lack of motivation and patients’ lack of awareness about the role of pharmacists. ^31^^–^^34^

All over the world, pharmacists are suffering from lack of time and increased workload. Therefore, pharmacists are unable to provide professional pharmaceutical care to every patient, which negatively affects patient satisfaction. This finding is consistent with a previous study conducted in UAE and other countries. ^31^^–^^34^

Most people in developing countries see pharmacists as drug dispensers because their interaction with pharmacists is limited to dispensing the prescription and medicines. ^7^^,^^35^ Pharmacists should promote pharmaceutical care by practicing it with their patients until this practice becomes routine and expected when patients visit a pharmacy. This will help raise awareness of the role of pharmacists in the health care system and in society. ^7^

These solutions should be supported by the health authorities as the adoption of this practice will have many positive effects on the health system of the country.

## Strength and limitation of the study

Although participants were randomly selected from the different emirates of the UAE, the sample selection was not systematically distributed. In fact, we had difficulty recruiting participants because there were many barriers to active participation by pharmacists, such as lack of time and an insufficient number of supporting staff. In addition, the number of participants from hospital pharmacies was low because most pharmacists work in community pharmacies. The current study has several strengths: The study had a representative sample of pharmacists who were randomly selected, the technique used for data collection (face-to-face written questionnaires and appointment interviews) had a high response rate, and two methods of analysis were used. Therefore, the results of our study can be generalized to represent a broader population.

This study suggests that pharmacists working in the UAE do not implement pharmaceutical care as part of their daily routine work. However, pharmacists in the UAE have a positive attitude towards pharmaceutical care but lack consistency in delivering these services. Pharmacists are well placed to practice pharmaceutical care and they can provide it to patients when needed, but this is not enough. The health care system needs more pharmacist engagement. Pharmacists need to bridge the gap between them and physicians because they share a common goal to improve patient outcomes and quality of life. Health authorities should pay more attention to the role of pharmacists in the health system and help them to overcome the obstacles and barriers in the spread of pharmaceutical care to facilitate pharmaceutical care in practice and see the positive impact of this practice in all areas.

The major barriers faced by pharmacists in the UAE are patients’ ignorance of the pharmacist’s role, lack of time, and lack of motivation to implement this concept. Therefore, concerted efforts involving health authorities and educational institutions should be made to transform this profession from its traditional practice to a more patient-centered practice.

## Data availability

### Underlying data

Open Science Framework: Current views of community and hospital pharmacists on pharmaceutical care services in the United Arab Emirates: A mixed methodological study. https://doi.org/10.17605/OSF.IO/XB5YZ. ^36^

This project contains the following underlying data:

‐ Full transcripts Qualitative interview.docx
‐ Responses.xlsx

### Extended data

Open Science Framework: Current views of community and hospital pharmacists on pharmaceutical care services in the United Arab Emirates: A mixed methodological study. https://doi.org/10.17605/OSF.IO/XB5YZ. ^36^

This project contains the following extended data:

- Questionnaire.pdf

## Reporting guidelines

- COREQ_checklist F1000R.pdf
- STROBE_checklist_cross-sectional score updated.doc

Data are available under the terms of Creative Commons Attribution 4.0 International License (CC-BY 4.0).
