# Supplementary material for: Current views of community and hospital pharmacists on pharmaceutical care services in the United Arab Emirates: A mixed methodological study
Source: F1000Res. 2022 Dec 1;11:694. Originally published 2022 Jun 23. [Version 2] doi: 10.12688/f1000research.110102.2 (PMC9745206; doi:10.12688/f1000research.110102.2)
Supplement: Peer review history

# Peer review: Reviewer response for version 2

**DOI:** 10.5256/f1000research.139986.r157138

**Published:** 2022-12-12

**Authors:** Hadeer Akram Abdulrazzaq Al-Ani

**Affiliations:** 1 Department of Public Health, School of Medicine, University of California Davis, Davis, CA, USA

Dear Authors,

 I thank you for the corrections I asked for in the first round. I congratulate you on this achievement and grant you the approval.

 My best regards

Is the work clearly and accurately presented and does it cite the current literature?

Yes

If applicable, is the statistical analysis and its interpretation appropriate?

Yes

Are all the source data underlying the results available to ensure full reproducibility?

Yes

Is the study design appropriate and is the work technically sound?

Yes

Are the conclusions drawn adequately supported by the results?

Yes

Are sufficient details of methods and analysis provided to allow replication by others?

Yes

Reviewer Expertise:

Clinical Pharmacy, Public Health, Biostatistics, Genetic pharmacology, and Pharmacoepidemiology.

I confirm that I have read this submission and believe that I have an appropriate level of expertise to confirm that it is of an acceptable scientific standard.

# Peer review: Reviewer response for version 2

**DOI:** 10.5256/f1000research.139986.r157139

**Published:** 2022-12-02

**Authors:** Diana Malaeb

**Affiliations:** 1 College of Pharmacy, Gulf Medical University, Ajman, United Arab Emirates

I read the authors' responses made to my comments. I can see that all my comments have been addressed by the authors. I now can consider that Manuscript is approved for indexing.

 Best wishes

Is the work clearly and accurately presented and does it cite the current literature?

Yes

If applicable, is the statistical analysis and its interpretation appropriate?

Yes

Are all the source data underlying the results available to ensure full reproducibility?

Yes

Is the study design appropriate and is the work technically sound?

Yes

Are the conclusions drawn adequately supported by the results?

Yes

Are sufficient details of methods and analysis provided to allow replication by others?

Yes

Reviewer Expertise:

Clinical practice

I confirm that I have read this submission and believe that I have an appropriate level of expertise to confirm that it is of an acceptable scientific standard.

# Peer review: Reviewer response for version 1

**DOI:** 10.5256/f1000research.121677.r141860

**Published:** 2022-09-15

**Authors:** Diana Malaeb

**Affiliations:** 1 College of Pharmacy, Gulf Medical University, Ajman, United Arab Emirates

This is a mixed methodological study among hospital and community pharmacists across the UAE. The aim of the study is to explore the current views of pharmacists in the United Arab Emirates (UAE) on pharmaceutical care services and the nature of barriers encountered in practice using qualitative and quantitative assessment methods. The study has adequate power and used a validated tool in quantitative (n=305 pharmacist) and qualitative (n=15 interviewer pharmacists) approaches. However, there are few points that the authors need to clarify and elaborate more:

  

1. The title of the manuscript is explicit and covers the scope of the study. The method is appropriate, but the study need to include more number of hospital pharmacists to be consistent with the number of community pharmacists. I suggest this need to be clearly added in the limitation part of the study.
2. The seven criteria that the questionnaire was consisted of was wide enough to cover many important aspects in pharmaceutical care services as I think; these are 1) :vital statistics of the population, 2) pharmacist-physician interactivity, 3) pharmacist’s role and involvement in patient counseling, 4) handling and reporting of adverse drug reactions in patients, 5) participation in health awareness programs, 6) pharmacist’s role in minimizing prescribing errors and identifying drug-related problems, 7) barriers to implementing pharmaceutical care in the UAE. Authors need to briefly explain the selection of these seven barriers among other barriers and suggest few other barriers that future studies need to explore.
3.  The response rate was 76% (305/400 invited pharmacists)! Authors need to give reasons for the low response rate.
4. There is one table that describes the demographics.  However, I did find it very interesting that the number of participants aged>50 is only 5 participants, can the authors elaborate more on that?
5. I see that there are statistical differences in table-4 (Patients inform the pharmacists about ADRs occurrence and ask about how it can be managed---but does that translate into actual differences? Does it?
6. The finding in table-4 (Patients are aware of ADRs reporting) need to be discussed more in the discussion part. How can you explain the type of pharmacy and patients awareness of ADR?
7. I enjoyed reading the manuscript and found it interesting--- but was surprised about the barriers to implementing pharmaceutical care in the UAE! 63.3% of patients think they are not interested or in a hurry and 32% of them thought that the professional role of pharmacists is not identified or clear. I think the authors covered these points in the discussion but they need to explain more why these two barriers existed and suggest solutions for them.

Is the work clearly and accurately presented and does it cite the current literature?

Yes

If applicable, is the statistical analysis and its interpretation appropriate?

Yes

Are all the source data underlying the results available to ensure full reproducibility?

Yes

Is the study design appropriate and is the work technically sound?

Yes

Are the conclusions drawn adequately supported by the results?

Yes

Are sufficient details of methods and analysis provided to allow replication by others?

Yes

Reviewer Expertise:

Clinical practice

I confirm that I have read this submission and believe that I have an appropriate level of expertise to confirm that it is of an acceptable scientific standard, however I have significant reservations, as outlined above.

# Response

**Published:** 2022-10-17

**Authors:** Zelal Kharaba

**Affiliations:** Alain University, United Arab Emirates

**Author**: This is a mixed methodological study among hospital and community pharmacists across the UAE. The aim of the study is to explore the current views of pharmacists in the United Arab Emirates (UAE) on pharmaceutical care services and the nature of barriers encountered in practice using qualitative and quantitative assessment methods. The study has adequate power and used a validated tool in quantitative (n=305 pharmacist) and qualitative (n=15 interviewer pharmacists) approaches. However, there are few points that the authors need to clarify and elaborate more:

- The title of the manuscript is explicit and covers the scope of the study. The method is appropriate, but the study need to include more number of hospital pharmacists to be consistent with the number of community pharmacists. I suggest this need to be clearly added in the limitation part of the study.

**Response**: We would like to thank the reviewer for her time and efforts in critically reviewing our manuscript. Please note that the suggested comment is added to the limitation part of the study and highlighted in yellow.

- The seven criteria that the questionnaire was wide enough to cover many important aspects in pharmaceutical care services as I think; these are 1) :vital statistics of the population, 2) pharmacist-physician interactivity, 3) pharmacist’s role and involvement in patient counseling, 4) handling and reporting of adverse drug reactions in patients, 5) participation in health awareness programs, 6) pharmacist’s role in minimizing prescribing errors and identifying drug-related problems, 7) barriers to implementing pharmaceutical care in the UAE. Authors need to briefly explain the selection of these seven barriers among other barriers and suggest few other barriers that future studies need to explore.

**Response:** We would like to thank the reviewer for her time and efforts in critically reviewing our manuscript. Please note that the selection of the previous aspects was after an extensive literature review of many articles that deeply discussed many aspects in pharmaceutical care services. These aspects were gathered in the study questionnaire which was validated and piloted. We think that future studies need to explore these barriers in line with COVID-19 pandemic. In addition, future studies need to focus also on te effective participation of hospital pharmacists in reporting medication errors by going through several steps, namely: consistently reporting errors, collecting the reports monthly, analyzing the reports based on the nature of the errors, providing solutions on how to avoid them in the future, then sharing and discussing the errors and solutions in meetings with the hospital medical team to raise awareness and prevent such errors.

  

-  The response rate was 76% (305/400 invited pharmacists)! Authors need to give reasons for the low response rate.

**Response: **We would like to thank the reviewer for her time and efforts in critically reviewing our manuscript. In fact the low response rate among pharmacists is attributed to their busy schedule, the unavailability of sufficient number of pharmacists and the lack of appropriate time.

  

- There is one table that describes the demographics.  However, I did find it very interesting that the number of participants aged>50 is only 5 participants, can the authors elaborate more on that?

**Response: **In fact, UAE considered as a youth country with more than 77% of its population aged less than 55 years of age according to the statistics center 2022 ( https://www.globalmediainsight.com/blog/uae-population-statistics/). This is applied for the pharmacy sectors as well.

  

- I see that there are statistical differences in table-4 (Patients inform the pharmacists about ADRs occurrence and ask about how it can be managed---but does that translate into actual differences? Does it?

**Response: **We would like to thank the reviewer for her time and efforts in critically reviewing our manuscript. In fact this is translated into actual references as 62% of participants received patients report of ADRs.

  

- The finding in table-4 (Patients are aware of ADRs reporting) need to be discussed more in the discussion part. How can you explain the type of pharmacy and patients awareness of ADR?

**Response: **We would like to thank the reviewer for her time and efforts in critically reviewing our manuscript. Table 4 shows the patients' reports of adverse drug reactions that occurred and the pharmacists' responses. In detail, the community pharmacists showed significantly a higher level of patients’ awareness of adverse drug reaction reporting than hospital pharmacists.

- I enjoyed reading the manuscript and found it interesting--- but was surprised about the barriers to implementing pharmaceutical care in the UAE! 63.3% of patients think they are not interested or in a hurry and 32% of them thought that the professional role of pharmacists is not identified or clear. I think the authors covered these points in the discussion but they need to explain more why these two barriers existed and suggest solutions for them.

**Response: **We would like to thank the reviewer for her time and efforts in critically reviewing our manuscript. We think that the main cause of that is related to the long time consumed by patients in the medical centers and hospitals which affects the pharmacists’-patients’ discussion time. We suggest that the waiting time in health care centers need to be reduced and more time for the patients to spend with the pharmacists.

# Peer review: Reviewer response for version 1

**DOI:** 10.5256/f1000research.121677.r141859

**Published:** 2022-07-11

**Authors:** Hadeer Akram Abdulrazzaq Al-Ani

**Affiliations:** 1 Department of Public Health, School of Medicine, University of California Davis, Davis, CA, USA

I would like to thank the authors for sharing their work in writing this manuscript. I have minor corrections which need to be edited/corrected. Ambiguous results should be detailed because everything should be clear to readers.

1. Please update all references within the last five years (2017-2022), especially those related to the problem statement, methodology, and discussion sections. The majority of references are outdated.
2. In **Statistical Analysis**: Please correct the Chi-square to find the association, not the correlation. “A Chi-square test was used to determine the significant correlation between..”
3. **Results**. Rephrase “There was a statistically significant association between patients’ reports of adverse drug reactions and pharmacist age (p = 0.0216) and type of pharmacy (p = 0.0482)”. “There was a statistically significant relationship between participation in health awareness programs and pharmacists’ age (p = 0.0441, p = 0.0002) and years in practice (p = 0.042)”.
4. **Results**. Table 3. Please give more explanation in relevant texts for the results which type of pharmacy (community or hospital) showed a higher amount of time. Same thing for informing the patients about drug side effects and years of experience. Need explanation about “Physicians are ready to accept the pharmacists’ participation” and years of experience.
5. **Results**: Table 4. Please provide more details in relevant texts about which pharmacy showed a higher level of patient awareness of ADRs reporting.
6. **Results**: Table 5. Please give more details in relevant texts about which age with hospital organizes health awareness programs and pharmacists' participation in organizations. Same for the experience with the hospital organizes health awareness programs and pharmacists' satisfaction.
7. **Results**: Table 6. Please give more details in relevant texts about which age and years of experience are more levels of irrelevant drug prescriptions
8. Figure 1. Please select another color than black (like white) for the percentage in bars. It is not clear.
9. **Discussion**: “The current results were consistent with several previous studies”. Which studies?
10. Some sentences in the discussion are without references. Please add more references. Like “These incorrect assumptions are due to the pharmacists’ lack of motivation and patients’ lack of awareness about the role of pharmacists”

Is the work clearly and accurately presented and does it cite the current literature?

Yes

If applicable, is the statistical analysis and its interpretation appropriate?

Yes

Are all the source data underlying the results available to ensure full reproducibility?

Yes

Is the study design appropriate and is the work technically sound?

Yes

Are the conclusions drawn adequately supported by the results?

Yes

Are sufficient details of methods and analysis provided to allow replication by others?

Yes

Reviewer Expertise:

Clinical Pharmacy, Public Health, Biostatistics, Genetic pharmacology, and Pharmacoepidemiology.

I confirm that I have read this submission and believe that I have an appropriate level of expertise to confirm that it is of an acceptable scientific standard, however I have significant reservations, as outlined above.

# Response

**Published:** 2022-10-17

**Authors:** Zelal Kharaba

**Affiliations:** Alain University, United Arab Emirates

Thank you for taking the time to review our manuscript and for your valuable comments. Kindly find here our point-to-point reply to your comments:

  

- Please update all references within the last five years (2017-2022), especially those related to the problem statement, methodology, and discussion sections. The majority of references are outdated.

**Response: **We would like to thank the reviewer for his time and efforts in critically reviewing our manuscript. Please note that References have been updated as per the advice, however, we kept few references- although outdated - as they are important for the idea of our article. 

  

- In **Statistical Analysis**: Please correct the Chi-square to find the association, not the correlation. “A Chi-square test was used to determine the significant correlation between..”

**Response: **Revised and highlighted in the text.

  

- **Results**. Rephrase “There was a statistically significant association between patients’ reports of adverse drug reactions and pharmacist age (p = 0.0216) and type of pharmacy (p = 0.0482)”. “There was a statistically significant relationship between participation in health awareness programs and pharmacists’ age (p = 0.0441, p = 0.0002) and years in practice (p = 0.042)”.

**Response: **Revised and highlighted in the text.

  

- **Results**. Table 3. Please give more explanation in relevant texts for the results which type of pharmacy (community or hospital) showed a higher amount of time. Same thing for informing the patients about drug side effects and years of experience. Need explanation about “Physicians are ready to accept the pharmacists’ participation” and years of experience.

**Response: **We would like to thank the author for his insightful comment which will improve our article. More explanation were added to the text as advised (highlighted in yellow).

 To detail the patient counselling approach (Table 3), there was a significant association between the amounts of time spent with each patient in community pharmacy compared to the hospital pharmacy. Additionally, pharmacists who practiced more than 10-years are significantly more engaged in patient counselling which reflect a higher physician acceptance rate as well as more patient education about side effects.

  

- **Results**: Table 4. Please provide more details in relevant texts about which pharmacy showed a higher level of patient awareness of ADRs reporting.

**Response: **We would like to thank the reviewer for drawing our attention for this point. More explanation were added to the text as advised (highlighted in yellow).

 In detail, the community pharmacists showed significantly a higher level of patients’ awareness of adverse drug reaction reporting than hospital pharmacists.

- **Results**: Table 5. Please give more details in relevant texts about which age with hospital organizes health awareness programs and pharmacists' participation in organizations. Same for the experience with the hospital organizes health awareness programs and pharmacists' satisfaction.

**Response: **We would like to thank the reviewer for drawing our attention for this point. More explanation were added to the text as advised (highlighted in yellow). In details, there was a significant difference among the older age pharmacists and the participation in health awareness program and the organization in such program. In addition, pharmacists who have more years of experience showed more satisfaction toward the health awareness programs.

  

- **Results**: Table 6. Please give more details in relevant texts about which age and years of experience are more levels of irrelevant drug prescriptions

**Response: **We would like to thank the reviewer for drawing our attention for this point. More explanation were added to the text as advised (highlighted in yellow). In details, pharmacists with more years of practice showed to be more active in identifying any irrelative drug prescription.

- Figure 1. Please select another color than black (like white) for the percentage in bars. It is not clear.

**Response: **Revised.

  

- **Discussion**: “The current results were consistent with several previous studies”. Which studies?

**Response: **Revised and references added to this sentence.

- Some sentences in the discussion are without references. Please add more references. Like “These incorrect assumptions are due to the pharmacists’ lack of motivation and patients’ lack of awareness about the role of pharmacists”

**Response: **Revised and references added.
